# Politics or markets: The dual role of the motivation to achieve organizational legitimacy in the development of knowledge management capabilities and business model innovation

**DOI:** 10.3389/fpsyg.2023.1112240

**Published:** 2023-05-23

**Authors:** Suqin Liao, Jingjing Wei, Qianying Hu

**Affiliations:** School of Management, Zhejiang University of Technology, Hangzhou, Zhejiang, China

**Keywords:** market legitimation motivation, political legitimation motivation, knowledge management capabilities, business model innovation, new venture

## Abstract

Despite business model innovation being the object of much interest, limited attention has paid on how and when knowledge management capabilities enhance business model innovation in the literature. Build upon institutional theory and knowledge-based view, we seek to investigate how knowledge management capabilities affect the business model innovation by exploring the dual role of different types of legitimation motivations in triggering knowledge management capabilities, and moderating the relationship between knowledge management capabilities, and business model innovation. The data collected from the 236 Chinese new ventures running their businesses across a variety of sectors. The results indicate the both political and market legitimation motivation positively affect knowledge management capabilities. The relationship between knowledge management capabilities and business model innovation are more strongly in high motivation to achieve market legitimacy. However, the positive effect of knowledge management capabilities stimulate business model innovation is more strongly in moderately motivation to achieve political legitimacy than in low or highly political legitimation motivation. The paper has significantly contributed to advancing the body of knowledge of institutional and business model innovation theory and providing deeper insights on the correlation between firm’s motivation to achieve legitimacy and knowledge management capabilities for business model innovations.

## Introduction

In the specific context of start-ups, business model innovation (BMI) is perceived to have a pivotal role pursuing a higher level of efficacy and competitive advantage (Chesbrough, 2010). Examples of BMI include Netflix, which innovated the business model by providing online movies rental services (Snihur and Zott, 2020), or Pinduoduo, which offers cost-effective products from online group purchasing sites. Given the importance of BMI for new ventures’ long-term viability, previous research raises a number of crucial theoretical and empirical questions: what are the drivers, facilitators, and hindrances of the BMI? (Foss and Saebi, 2017). In addition to the external factors, such as environmental dynamism, technology advances (Lehoux et al., 2014), an important internal motivation for BMI in new ventures that previous studies have been examined is the knowledge management capabilities (KMCs) (Hock-Doepgen et al., 2021). KMCs refers to the firm’s capabilities to reconfigure and realign the process of knowledge exploration, exploitation, and retention across functional boundaries (Teece, 2018). Previous studies suggest that KMC can identify or generate valuable ideas, develop and commercialize the new ideas to extract value, and then facilitate the BMI (Teece, 2018). While past research has thoroughly investigated the contribution of KMCs in the context of BMI, our understanding of how do the KMCs contribute to the BMI remains limited.

The institutional-based view suggests that knowledge reflects an understanding of how an institutional system operates and how institutional rules work (Zott and Huy, 2007). Separating knowledge from the institutional environment is difficult, thus suggesting that knowledge itself is institution-dependent. An important institutional motivation for BMI in new ventures that previous studies have been examined is the desire to attain legitimacy (Zott and Huy, 2007). Legitimacy generally refers to “a generalized perception or assumption that the actions of an entity are desirable, proper, or appropriate within some socially constructed system of norms, beliefs, and definitions” (Suchman, 1995). It is often regarded as a resource that is available to actors in the focal business ecosystem for their own opportunistic purpose (Press et al., 2020). When a firm yields a motivation to gain legitimacy to convince external stakeholders, its approach in integrating new knowledge and adopting a new practice tends to be fast (Cheng, 2010). For instance, the pursuit of the ISO standard suggests that the firms adhere to establishing a quality management system (Kannan-Narasimhan, 2014), which helps firms increase efficiency, cut costs, and allow them to progress their innovation idea toward to development (Fisher et al., 2017). Based on this argument, the legitimation motivation would seem to moderate the effect of KMCs and BMI. However, previous studies also indicate that the KM as part of organizational structures and processes, have to be congruent with wider social and normative practices embedded in the institutional environment. The effectiveness and efficiency of knowledge creation, transmission, and relocation is partly determined by the institutional-based activities (Lu et al., 2008). Thus, the legitimation motivation may also act as antecedent to KMCs.

In addition, to acquire resource from external stakeholders and government to achieve sustainable development, new ventures often seek to meet rules devised by the government and interplay with market players (Guo et al., 2018). Therefore, based on the two critical groups of audiences who judge legitimacy, organizational motivation to be seen as legitimate can be divided into market legitimation motivation (MLM) and political legitimation motivation (PLM). As different audiences hold different interests, and, in turn, evaluate legitimacy (Bunduchi, 2017), the motivations to achieve different types of legitimacy will exert different influences on organizational change. However, to the best of our knowledge, there exists limited empirical evidence that take the idiosyncrasies of different types of legitimation motivation and investigates its contribution for KM and innovation strategy.

To address the previously mentioned question theoretically and empirically, we proposed an integrated theoretical framework to illustrate how the KMCs affect BMI by analyzing the dual impact (antecedents and moderators) of two important motivations to achieve organizational legitimacy (PLM and MLM). Our main theoretical insights consist of three parts. First, we show that KMCs is driven by the level and type of a firm’s motivation to achieve legitimacy, and it is not immediately beneficial for BMI, but the value is also contingent on the level and type of a firm’s legitimation motivation. Second, we show that legitimation motivations perform a dual role: they facilitate the KMCs within the organization and shape the impact of KM practices on BMI. The findings draw a more comprehensive picture of the organizational legitimation motivation behind a certain action, thereby laying down a threshold over which to elaborate the implications of motivation to be legitimate. Third, we identifying two distinct types of legitimation motivation and show that they have different consequences. We show that inter-firm heterogeneity in legitimacy motivation trigger difference in the benefits from KM practices. Fourth, this study adds knowledge to BMI literature by integrating knowledge-based view (KBV) and institutional theory in the field of entrepreneurship and the context of the emerging economy.

## Theoretical framework and literature review

### The KBV and institutional-based perspective for conceptual framework

As a theoretical extension of resource-based view (RBV), KBV conceptualizes the firm as an institution that develop and integrate value-creating knowledge (Kim et al., 2014). The differences between the firms’ possession of not only the knowledge but also the capabilities of knowledges management have significant effects on their core competencies. From this perspective, organizational knowledge or information as a resource that need to be managed consistently, and it considered as the main source of innovating strategies and sustaining competitive advantage (Inkinen, 2016; Rehman et al., 2021). Penco (2015) suggested that the firms in knowledge economy have a greater reliance on intangible assets such as knowledge rather than on physical resources. Therefore, the knowledge management within an organization can be a focus point for sustained value creation (Alavi and Leidner, 2001).

However, the KBV alone may not be very effective all the way, and the knowledge must be perceived as legitimate and has to fit institutional requirements. Failure to incorporate the institutional factors into the KBV may lead to a flawed view that knowledge-based advantages are not constrained by the institutional force in which they are employed (Brouthers et al., 2008; Lu et al., 2008). The institutional theory has been identified as the most important perspective to understand organizational legitimacy. Institutional theory primarily explores the role of social influence and pressures in shaping organizational behavior and performance (DiMaggio and Powell, 1983; Press et al., 2019). Campbell (2004) indicated that: “Institutions are the foundation of social life. They consist of formal and inform rules, monitoring and enforcement mechanisms, and systems of meaning that define the context within which individuals, corporations, labor unions, nation-states, and other organizations operate and interact with each other.” That is to say the institutional environment can affect the firm’s strategic choice and capabilities (Tsinopoulos et al., 2018), and the firms’ activities and capabilities are also motivated by the longing to be consistent with established cognitive structure in society (e.g., rules and regulations) (Oliver, 1997). Thus, the effectiveness and efficiency of knowledge creation, transmission, and integration is partly determined by the institutional force (Lu et al., 2008; Liu et al., 2018). In addition, institutional theory also highlights the importance of institutional force in shaping the development of product innovation strategies in emerging economies (Jean et al., 2014).

Previous studies has argued that institutional theory can be adopted to investigate the firms’ strategic behaviors (Bruton et al., 2010; Donbesuur et al., 2020). However, empirical evidence on integrating KBV and institutional theory to examine firms’ KM practice and innovation behavior is still limited. Combining the KBV with institutional theory encourages the perspective that the institutional factors will directly affect the KM and also can affect the influence of the use of knowledge on innovate strategies. Thus, the KBV and institutional theory represents the suitable framework for investigating how KMCs be leveraged to facilitate BMI (innovation strategies) and exploring the possible enabler and moderate of specify motivation to achieve legitimacy (institutional force).

### KMCs and BMI in new venture

A business model refers to a framework for a firm’s boundary-spanning transaction with an external business participant. It depicts the content, structure, and governance of transaction designed to create value (Amit and Zott, 2012). BMI involves implementing a new business model to the firms (Chesbrough, 2010; Velu, 2015). Such innovation has been confirmed as a key factor for firms to acquire competitive advantage by innovating business model (Velu, 2015; Zheng et al., 2020). BMI can be introduced by both new ventures and established firms, but most often occurs in new ventures or at an early stage of an enterprise’s development (Foss and Saebi, 2018). For example, Lawton et al. (2007) explain BMI occurs in three new ventures, including Netflix, Costco, and eBay. New ventures have fewer stakeholders, thus making them more flexible and less path-dependent to switch business models (Parker and Van Praag, 2012). However, BMI in new venture also faces unpredictable challenges that they are relatively weak in resources and knowledge (Sainio et al., 2011), they have difficulty obtaining appropriate values from recognized opportunities, thereby hindering BMI (Pucci et al., 2017; Liao et al., 2019).

Based on the argument of KBV that firm’s existing knowledge base delimits its scope and capacity to comprehend and apply novel knowledge to radical innovations (Inkinen, 2016), studies provide evidence that KMCs can help new venture to innovate the business process (Du Plessis, 2007; Wu et al., 2013; Hock-Doepgen et al., 2021). Previous literature often define KM as the process with the aim to reuse, awareness, and to share and learn the knowledge across the organization through identifying, acquiring, transferring and diffusing the knowledge (Du Plessis, 2007; Kamhawi, 2012). In this sense, KM involves the structures and practices that firms use to aggregate, integrate, and employ knowledge as leverage appropriately, which can facilitate entrepreneurship within the organization (Bhardwaj, 2019). KMCs aim to unleash its intellectual potential through increasing the effectiveness of the management of organizational knowledge resources (Jordão and Novas, 2022; Zhan and Xie, 2022). A firm focused on the development and of knowledge provides its human capital with rapid access to requested knowledge and technologies (Palacios et al., 2009). Therefore, new ventures with strong KMCs can avoid considerable challenges of BMI, including limited key resources (Zheng et al., 2020), burdens from the experimentation (Chesbrough, 2010), and the unchangeable inertia of founders (Cardon et al., 2009). For instance, Darroch (2005) suggest that a firms capable in all three KM components is more innovative, and different customer-related knowledges contribute to value creation in BMI within different mechanisms (Wu et al., 2013).

The previous studies has advanced our understanding about the enabling role of KMCs on BMI (Wu et al., 2013; Yu et al., 2020). However, so far, there is limited studies have empirically explore through what mechanism do KMCs facilitate BMI. We considered this is an important omission. Although Hock-Doepgen et al. (2021) have examined the impact of internal and external KMCs on BMI and how organizational risk-taking tolerance moderated theses direct effects, empirically analyses simultaneously exploring the enabler role of KM and the institutional moderating role between KMCs and BMI must still be done.

### The motivation to achieve organizational legitimacy

According to the KBV and institutional theory, BMI in new ventures is the result not only of combining and coordinating external resources to gain and internalize new knowledge, but also of legitimizing and conforming to normative standards and pressures. As part of organizational structures and processes, KM practices and innovation strategies must be aligned with social and normative practices embedded in the institutional environment (Liu et al., 2018). Legitimacy is “a generalized perception or assumption that the actions of an entity are desirable, proper, or appropriate within some socially constructed system of norms, values, beliefs, and definitions” (Suchman, 1995: 574). Thus, the motivation to achieve organizational legitimacy will encourage the firms to share beliefs about the normal behavior such as, forging ties (Bohnsack et al., 2014; Fisher et al., 2017), engaging in symbolic actions (Zott and Huy, 2007), and developing new knowledge to make a business plan (Karlsson and Honig, 2009). These initiative activities will strengthen the organizational learning culture, enhance the understanding and further refinement of existing knowledge, and also encourage the opening up to acquire external ideas, which could facilitate the experiment for new alternatives (Oeij et al., 2017; Tsinopoulos et al., 2018). Therefore, the legitimation motivation may serve as a key factors to facilitate KM practices and innovation strategies. While the role of organization legitimacy in allowing greater access to knowledge and resources has been acknowledged in some studies (Gong, 2006; Lu et al., 2008), the relationship between the motivation to achieve organizational legitimacy and KM has largely neglected in subsequent studies.

In addition, legitimacy refers to the social judgment in the eyes of the stakeholders, and thus the assessment of legitimacy is dependent on the audience. Prior research on institutional logic suggests that there are two types of legitimate audiences: market players (e.g., investor, customer, and other market resource providers) and political subjects (e.g., government and agency) (Fisher et al., 2017). The logic behind these two types of legitimacy audiences is different, and therefore the criteria for judging the organizational legitimacy are different (Thornton and Ocasio, 1999). Political subjects are judged by whether the enterprise can bring political or public benefits, while the estimate of market subjects is whether the business is successful or not. Numerous transition economies have made remarkable economic progress that is attributable to their market-oriented reforms and government support (Zheng et al., 2014). Both the market and political forces are vital to the firm’s development in the context of institutional transformation (Marquis and Raynard, 2015). Herein, we unpack the motivation to achieve organizational legitimacy into market legitimation motivation (MLM) and political legitimation motivation (PLM).

Market legitimation motivation refers to the degree to which a firm’s desire to access the recognition and acceptance of the market audience, such as customers, suppliers, or competitors. PLM stresses the motivation to receive recognition and support from the government. Firms with MLM are inclined to take actions that enhance their competitiveness, such as being sensitive to environmental change and sustaining an organizational learning culture (Guo et al., 2018). To establish and maintain political legitimacy, firms will conduct behaviors to comply with the government’s norms and laws, such as supporting sustainable social development (Okhmatovskiy, 2010).

**FIGURE 1:**
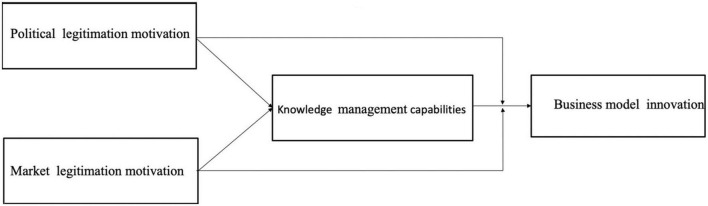
Theoretical research model.

Using institutional theory, scholars have provided insights into how organizational legitimacy work to affect the innovation strategies and capabilities (Rao et al., 2008). However, scant research examines the dual role (enabler and moderating) of two types motivation to achieve legitimacy on the KMCs-BMI nexus. Thus, to fill these gaps in the literature, we integrate KBV and institutional theory seek to explore the dual effects of PLM and MLM on the developing of KMCs and on the relationship between KM capabilities and BMI, respectively. Figure 1 shows the framework of this study. The theoretical framework show that how legitimation motivation influence the way in which the organizational capabilities to manage the knowledge, as well as how firms leverage the knowledge in an effort to innovate the transaction structure, content and governance.

## Hypotheses

### The antecedent role of the motivation to achieve organizational legitimacy

Knowledge management process comprises knowledge acquisition, knowledge sharing and knowledge application (Martinez-Conesa et al., 2017). We suggest that the legitimation motivations will facilitate the KMCs. First, prior studies suggest that the firms with desire to achieve legitimacy will encourage the firms actively enhance the density of inter-organizational contact and inter-organizational origin of knowledge as specific attributes to help firms maintain and enhance trust and reliability from the stakeholders (Dacin et al., 2007). These initiatives are likely to improve and enhance collaborative relationships between internal unit and partners of market and politic, which would increase internal and external novel knowledge acquisition, sharing, and application (Maurer, 2010). Second, increased motivation to adhere to regulation and standards of good practice will encourage the organizations to open the boundary for external knowledge and idea. Such opening can facilitate the knowledge acquisition. In addition, the legitimation motivation of good practice implies that the firms is willing to experiment that need high levels knowledge sharing and application (Tsinopoulos et al., 2018). Therefore, the motivation to achieve legitimacy contribute to KMCs.

Hypothesis1: MLM has a positive relationship with KM capabilities.

Hypothesis 2: PLM has a positive relationship with KM capabilities.

### The moderating role of the motivation to achieve organizational legitimacy

Knowledge management has a strong link to business strategy, as strong KMCs may access to new knowledge pools and valuable information, including the knowledge about new trends in technology and information on competitors (Zhou and Li, 2012). The new knowledge and critical information are vital for BMI because they are broad in scope and can provide insights to capitalize on various environmental trends effectively, increase the recognition for new business model opportunities, and promote the experimentation for novel business models (Hock-Doepgen et al., 2021). Therefore, we regards the positive relationship between KMCs and BMI as the baseline hypothesis.

### The moderating role of market legitimation motivation

We expect that MLM has an positive moderating effect on the relationship between KMCs and BMI. First, the encouragement of organizational learning and opportunities recognition that would result from the combination of KMCs and MLM. Meeting the standards of the market requires engagement with external parties (Tsinopoulos et al., 2018). Such engagement may increase the frequency and effectiveness of information exchange, and enhance the willingness to learn from the mistake (Zimmerman and Zeitz, 2002). Second, when the desire to achieve market legitimacy increases, managers are encouraged to take advantage of the information and knowledge that emerge from KMCs (Press et al., 2019), and will thus strengthen new firms’ ability to capture the latest trends of industry (market or technology trends). Such knowledge about development trend helps new venture to capture the business opportunities and take the preemptive innovation opportunities (Zimmerman and Zeitz, 2002). Third, adhere to the standards of market will restrain the opportunistic behavior of new ventures and encourage them to pursue sustainable development (Ganesan, 1994), which will encourage the willing to integrate and utilize the resource to seize market opportunity, trial and error, and innovation. Therefore, the following hypothesis is posed:

Hypothesis 3: MLM will positively moderate the relationship between KMCs and BMI.

### The moderating role of political legitimation motivation

We also expect PLM to have a positive moderating effect on the relationship between KMCs and BMI. First, meeting regulatory requirements and legislation of government can provide a fertile environment for learning. To satisfy the political and public interests in emerging economy, firms may change the technical core of the business, and, subsequently, contribute to organizational capabilities for learning (Martínez-Costa et al., 2008). For example, Bai et al. (2019) argued that motivation to establish political ties with the government facilitates communication between firms and government officials. In the communication process, firms gain insight into the institutional environment, and learn to act in accordance with the requirements of the government, which result in a fertile environment of learning and experimentation (Tarraço et al., 2021). Second, in order to establish and strengthen the political ties with the government, new ventures are often encouraged to expand the market (Sheng et al., 2011). Such expand process may reduce the perceived risk of change and increase the effectiveness of the resource utilization. As a result, the PLM has a positive moderating effect on the BMI of new venture. Therefore, we posit:

Hypothesis 4: PLM will positively moderate the relationship between KMCs and BMI.

## Methods and data

### Survey development

In this study, we used a questionnaire-based survey method to investigate the research model. Survey is a method that can enable generalizability outcomes, allow for easy replication, and help for the simultaneous investigation of a large number of factors (Pinsonneault and Kraemer, 1993). Additionally, suggestion by Straub et al. (2004), questionnaire-based survey research is a well-documented for accurately capturing the general tendency and identifying associations between constructs in a sample. Following the suggestion of Gerbing and Anderson (1988), existing measurement scales for the questionnaire items were identified based on a literature review and all measures were adapted from existing measure scales [e.g., (Guo et al., 2015)]. For new scales, measurement items were generated through a literature review followed by several tests such as professional review, content validity, and scale validity assessments.

To accomplish the object of this study, we developed an English version of the questionnaire following the prior research, and then, four independent translators translated it into Chinese and back-translated into English twice to ensure conceptual equivalence. Then, we modified the questionnaire according to the Chinese actual conditions. Before conducting the actual survey and following discussions on the proposed questionnaire, we conducted a pre-test by requesting six Chinese new ventures executives and two academics professionals to verify the content, clarity, and wording of the items (DeVellis, 2016). On the basis of the pilot test, we further revised a few questionnaire items to enhance the clarity, and finalized the survey. To ensure the consistency, the questionnaire contained a basic definition of KMCs, motivation to achieve legitimacy and BMI. Respondents answered questions on all subjective measures with Likert-scale anchoring of 1 (very low or strongly disagree) to 5 (very high or strongly agree). The multi-item constructs were operationalized by the mean value of all items. Appendix lists the measurement items used to operationalize the constructs.

### Sample and data collection

The sampling started with a dataset of new ventures from the local governments of China. China is paying special attention to entrepreneurship and innovation and trying to stimulate industrial upgrading. There are a lot of business model designs and novel technological knowledge constantly emerging in China, which result in a thriving innovation environment. Therefore, using Chinese data allows us to test the proposition in emerging economies, which important for extending the research in various contexts. Furthermore, understanding the BMI and KMCs in China can provide more general insight for economies at similar development stages in the region.

In order to study innovation in new ventures, we needed high-level executives with adequate business tenure to answer our questions, because lower-level staff might know little about the BMI that a sensitive topic closely related to the firm’s strategy and competitive advantage. Some researchers question the validity of studies that rely on a single informant’s perceptions because the issues with subjectivity (Lant et al., 1992). However, managers can provide reliable information about the innovation practices of their firms is a generally accepted belief that little evidence contradicts it Snihur and Wiklund (2019). The approach of adopting one informant per organization for evaluating motivations and innovation practices in this study is supported when survey instruments are well designed and administrated [e.g., Laursen and Salter (2006), Terjesen and Patel (2017)].

The first stage of the data collection process involved conducting in-depth interviews with 10 new ventures in the China. The interviews helped explicate the conceptual domain of BMI and facilitated the development and refinement of the study’s conceptual model. The interviews were conducted in a semi-structured manner to allow the informants to express their views and opinions within the research questions of the study. We then employ a quantitative approach based on the findings of the exploratory study. Following the specification of the study’s conceptual framework and hypotheses, and in line with previous studies on BMI, we collected our data from the start-ups list of local government *via* simple random sampling (Guo et al., 2017; Ciampi et al., 2021). The data was gathered from several provinces or municipalities of China: Zhejiang, Jiangsu, Beijing, Shanghai, Anhui, Shenzhen and Guangdong. Given that these provinces are typical areas for entrepreneurship and innovation. With the help of the science and technology bureau of the local government, a survey was conducted with one key informant approach.

The data were collected in two rounds. The first round began in June 2019 and ended in February 2020. We randomly selected 1,500 start-ups from the list, and then members of the research team made an email inquiry with senior managers who were CEOs, vice presidents, or chief inspectors of sample firms, to request participation. A total of 429 firms were willing to participate in the survey. An online survey platform was adopted to conduct the survey, and we sent a survey assessing KM capabilities, PLM and MLM to the target respondents. The informants clicked the link that sent through the WeChat or email and completed the survey. We assessed respondents’ self-report knowledge of the firm’s knowledge management, organizational legitimacy, and innovation-related activities on five-point answer scales ranging from 1 (poor) to 5 (excellent). The means of 4.1, 4.25, and 4.19, respectively, indicated that the informants were well-informed. To improve the response rate, we sent a reminder after 1 month and 3 months. A total of 388 firms provide the usage response. The second round began in September 2021 and ended in January 2022. We asked the prior informants in the survey to assess the measure of BMI. This round retrieved 301 answers. To prevent missing observation, we checked the completeness of the response and imposed a cutoff. As a consequence, 65 responses were discarded because these responses are partially completed. A total of 236 completed responses were included into the analysis yielding a valid response rate of 15.7%.

### Measures and validation of constructs

To verify the hypotheses of this study, all variables in this study were measured using reliable and valid scales cited in relevant past studies. Appendix provides a summary of the scales used in this study. BMI was measured with seven items initially adapted from an established reflective scale of Zott and Amit (2010). This scale is particularly recommended when predicting the effects or drivers of BMI in an organizational setting (Guo et al., 2015). We measured BMI by accessing the newness of predefined core element of business model: the content, structure, and governance of transactions.

To capture the KM capabilities, we used the measurement items developed by Liao et al. (2011), Martinez-Conesa et al. (2017). These items gauge the extent to which the organization engages in or supports the three interdependent KM activities (knowledge creation, knowledge sharing, and knowledge utilization) across firm boundaries. Knowledge creation pertains to the creation of knowledge resources across functional boundaries. Knowledge sharing reflect the sharing and distribution of knowledge of individual function. Knowledge utilization defined as the utilization of knowledge across functional boundaries.

The respondents were requested to assess their legitimation motivation taking into account facets such as political legitimation motivation and market legitimation motivation. Based on the previous work of Guo et al. (2018), Tsinopoulos et al. (2018), political legitimation motivation was operationalized to measure the extent of the motivation to obtain the recognition of government’s norms and laws by using a three-item scale. Four items were adopted to access the market legitimation motivation, which asks the respondents to evaluate the motivation to obtain the recognition of customer, supplier, industry peers and retailers.

Several variables may affect both firms’ motivation to achieve legitimacy, dynamic capability, and strategy innovation. Therefore, individual-level, firm-level and industrial or environment-level control variables were controlled for this research. We controlled two individual-level variables: founder age and founder start-up experience. Founder age was coded as one for younger than 20 years old, two for 20–30, three for 30–40, four for 40–50, five for older than 50. Start-up experience of founders was captured by a dummy variable (one indicating the respondent had started a business before, zero otherwise). Firm age and firm size were controlled as firm-level variables. Firm age was calculated as the number of years since the firm’s foundation, and firm size was measured based on a firm’s total number of employees (ranging from 1 for firms with fewer than 10 employees to 5 firms with 100 or more employees). Two industrial-or environmental–level variables were controlled: industry sectors and environmental turbulence. Industrial sectors generally influence a broad spectrum of firms’ strategy activities, and both the response to legitimation and the influence of KMCs and BMI could be different for different sectors. Thus, we coded three industry sectors (one pertaining to this industry; zero otherwise). The industrial included Chemicals and Pharmaceuticals (IC1), New Material and Chemicals (IC2), and Telecommunications (IC3), using Computers and IT as the baseline. Environment turbulence was measured by the scale adapted form Jansen et al. (2006).

### The construct reliability and validation

The reliability (as shown in Appendix) of the construct was evaluated by testing the internal consistency and composite reliability (CR). The CR of the constructs were above 0.7, and the value of Cronbach’s alphas exceeded 0.6 (Moss et al., 1998). The results indicate acceptable internal consistency reliability (Perreault and Leigh, 1989). The validation of the constructs was carried out by conducting the additional tests of convergent validity and discriminating validity. The discriminant validity was estimated by four methods. We first performed the confirmatory factor analysis using Mplus version 7.4 to test the validity of latent constructs. As shown in Table 1, the baseline model fitted the data well: χ2 was significant (χ2 = 123.12, *p* < 0.01) and the relative χ2 provided an acceptable fit with a *t*-test of χ2/df = 1.93 (less than the threshold of three), the root means square error of approximation (RMSEA) = 0.051 suggested an acceptable model fit, being less than 0.07, as required, standardized root mean square residual (SRMR) = 0.057, being less than 0.08 (Bentler, 1990). The global of fit index (GFI) = 0.932, the comparative fit index (CFI) = 0.916, the Tucker-Lewis index (TLI), also known as the non-normal fit index = 0.901. All of the fit index showed accepted value, being higher than 0.9, as required (Bentler, 1990).

**TABLE 1:** Confirmatory factory analysis for discriminant validity.

	Model	χ 2/df	RMSEA	CFI	GFI	TLI	SRMR
Baseline model	Four factors	1.93	0.051	0.916	0.927	0.918	0.057
Model 1	Three factors	2.13	0.069	0.876	0.900	0.843	0.081
Model 2	Two factors	2.58	0.086	0.817	0.812	0.794	0.088
Model 3	One factor model	3.16	0.091	0.775	0.728	0.714	0.090

Appendix shows the item loadings. We then compared the correlations and square roots of average variance extracted (AVEs), and the results shows that the square root of the AVE for each construct was greater than its correlation with any other reflective construct (as shown in Table 2) (Fornell and Larcker, 1981). We then conducted chi-square difference tests that performed pair-wise tests with each pair of constructs. A constrained model (correlation fixed to 1) is compared with an unconstrained model (correlated estimated freely). In each pair, the chi-square of the constrained model is significantly different from the unconstrained model, which offers evidence for discriminant validity (Anderson and Gerbing, 1988). We further conducted a four-factor measurement model (alternative model strategy). The results (see Table 1) show that the four-factor model provides a significantly better fit than other models, indicating acceptable discriminant validity (Cheng and Shiu, 2008). Finally, the maximum shared values (MSV) of constructs were lower than AVE, ensures the discriminant validity. The convergent validity was evaluated through testing the values of AVE and the loading of all items. As shown in Appendix, all the AVEs values of all constructs surpasses the 0.50 level (Fornell and Larcker, 1981), and the factor loadings were highly significant (*P* < 0.01), ranging from 0.659 to 0.821. These results suggest satisfactory convergent validity.

**TABLE 2:** Correlation among the construct.

	M	SD	1	2	3	4	5	6	7	8	9	10	11	12
1. Size	3.93	1.431	NA	–	–	–	–	–	–	–	–	–	–	–
2. Age	3.63	0.818	0.415	NA	–	–	–	–	–	–	–	–	–	–
3. FOA	2.86	0.980	0.033	0.101	NA	–	–	–	–	–	–	–	–	–
4. FOE	0.37	0.483	-0.007	0.005	0.209	NA	–	–	–	–	–	–	–	–
5. IC1	0.25	0.360	0.037	0.194**	0.191**	0.042	NA	–	–	–	–	–	–	–
6. IC2	0.05	0.158	-0.011	0.008	0.125	0.211**	-0.069	NA	–	–	–	–	–	–
7. IC3	0.09	0.441	0.057	-0.021	0.119	0.378**	-0.133*	-0.050	NA	–	–	–	–	–
8. ED	4.061	0.442	0.007	0.009	-0.057	0.022	-0.037	-0.096	-0.30*	NA	–	–	–	–
9. KMC	4.117	0.524	0.111	0.076	-0.194*	-0.065	-0.055	0.219*	0.040***	0.439**	**0.732**	–	–	–
10. BMI	4.002	0.527	0.193*	0.099	−0.110	−0.032	−0.063	0.140*	−0.131*	0.418**	0.497**	**0.789**	–	–
11. MLM	4.147	0.497	0.128	0.120	−0.031	0.030	−0.096	−0.197*	0.010*	0.533**	0.388**	0.542**	**0.720**	–
12. PLM	4.039	0.621	0.191*	0.146*	0.020	0.050	−0.109	−0.068	−0.052	0.500**	0.470**	0.562**	0.308**	**0.717**

FOA, founder age; FOE, founder star-up experience; IC1, chemicals and pharmaceuticals; IC2, new material and chemicals (); IC3, telecommunications; ED, environmental dynamism; KMC, knowledge management capability; BMI, business model innovation; MLM, market legitimation motivation; PLM, political legitimation motivation. The diagonal (bold) with underlining is the square root of AVE. Off-diagonal elements are the correlations among constructs. ***p < 0.001; **p < 0.01; *p < 0.05 (two-sided test).

### Addressing potential bias in sample

We took procedural and statistical remedies to minimize potential bias. To ensure that this study has no non-response bias, we compare the early with late respondents in terms of age, size, and capital by the *T*-test. The results indicated no significant differences between the two groups (Armstrong and Overton, 1977). To tackle additional reliability issues related to single response bias, we asked 20 firms that had voluntarily provided their formation to ask a partner to complete the survey. We test the intra-correlation coefficients (ICC) for each variable. The results show that the ICC scores ranged from 0.81 to 0.92, indicating single manager provided reliable information.

To evaluate the magnitude of common method bias, several procedural methods were employed. First, we obtained measures of the predictor and criterion variables from different time. Second, Harman’ one-factor analysis was conducted on the items included in the regressions. The result indicates that the presence of multiple factors explaining 52.44% of the total variance, and the variance was evenly dispersed among the factors (Harman, 1976). Hence, common method bias makes no sense in the outcome. Third, followed recommended guidelines of Lindell and Whitney (2001), the marker variable test was employed applied. The measurement of firm ownership was included as a theoretically unrelated marker variable in the model. No notable difference was found between the pairwise correlations of the main constructs and the partial correlations including marker variable. Therefore, common method bias did not significantly affect the parameter estimates.

## Analyses and results

### Descriptive analysis

Table 2 presents the means, standard deviations, and the pairwise correlations between the main variables. The correlations among the main variables are generally low, with a maximum absolute value of 0.562. In order to alleviate the concerns about multicollinearity effects as they could seriously bias the coefficient estimates, all constructs were zero-centered before regression (Cohen and Cohen, 2003). The variance inflation factors (VIF) values on all predictor variables were calculated, and the results show that the maximum VIF of assigned to one of the main constructs was 3.44, well below the cut-off point of 10 (Nieto and Santamaría, 2010). Hence, the multicollinearity problem could not be a threat to our results.

### Hypotheses testing

We to understand the role of the KM capabilities, PLM and MLM in determining the probability of an organization to innovate the business model, we used a firm unit of analysis to form our estimator. We adopted hierarchical regression analysis, using SPSS version 23.0, to assess the explanatory power of each set of variables. Regression is preferred for statistical modeling where independent variables are expected to have a direct effect on the dependent variable. Furthermore, the incremental effect of adding variables to the model can be observed in the hierarchical regression model. The independent, moderating, and control variables were entered into regression step by step to show the robustness of estimates.

Table 3 presents an overview of the regression results for the proposed research model. The significance of estimates (t-statistics) is obtained by performing a bootstrap analysis with 1,000 resamples. Direct effects of control variables on KMCs and BMI have been examined through Model 1 and Model 3, which established a baseline against other models. The control variables are also repeated in all other models. The results in Model 1 and Model 3 pointed out the effect of Telecommunications industry (IC3) was positive and significant (β = 0.151, *p* < 0.05) with regard to KMCs, but non-significant for BMI (β = 0.097, *p* > 0.05). Founder age had a negative and significant effect on KMCs (β = -0.169, *p* < 0.05), but non-significant for BMI (β = -0.084, *p* > 0.05). They also demonstrated that the environmental turbulence are positively and significantly associated with both KMCs (β = 0.408, *p* < 0.001) and BMI (β = 0.493, *p* < 0.001).

**TABLE 3:** The regression predicting firm performance (*t*-values).

	Model 1	Model2	Model 3	Model 4	Model 5	Model 6
	**Knowledge management capabilities**		**Business model innovation**			
**Control variables**						
Firm size	0.094 (1.497)	0.044 (0.713)	0.189** (3.181)	0.120* (2.333)	0.121 (2.171)	0.113 (2.170)
Firm age	0.058 (0.907)	0.025 (0.404)	0.036 (0.600)	–0.015 (–0.278)	0.006 (0.116)	–0.016 (–0.298)
Founder age	–0169* (–2.181)	–0.187* (–2.532)	–0.084 (–1.157)	–0.085 (–1.342)	–0.068 (–1.000)	–0.081 (–1.264)
Founder start–up experience	0.091 (1.158)	0.070 (0.926)	0.97 (1.301)	0.068 (1.059)	0.074 (1.079)	0.071 (1.107)
IC1	–0.074 (–1.163)	–0.042 (–0.693)	–0.167** (–2.778)	–0.120* (2.336)	–0.121* (–2.196)	–0.123* (–2.374)
IC2	–0.046 (–0.762)	0.003 (0.051)	–0.075 (–1.311)	0.005 (0.094)	–0.014 (–0.260)	–0.006 (–0.124)
IC3	–0.151** (–2.439)	–0.124* (–2.061)	–0.097 (–1.688)	0.006 (0.106)	–0.064 (–1.164)	–0.007 (–0.131)
Environmental turbulence	0.408*** (7.068)	0.243*** (3.597)	0.493*** (9.048)	0.166** (2.833)	0.264*** (4.413)	0.165** (2.799)
**Main effect**						
KM capability	–	–	–	0.212*** (3.698)	0.258** (3.523)	0.229** (3.315)
H1 →MLM	–	0.308*** (4.215)	–	0.341*** (5.313)		0.341*** (5.265)
H2 →PLM	–	0.130* (2.325)	–	0.169* (2.613)	0.152* (1.931)	0.144^+^ (1.912)
**Moderate effect**						
H3 →KM capability × MLM	–	–	–	0.136* (1.989)		0.168* (2.322)
H4 →KM capability × PLM	–	–	–	0.077^+^ (1.956)	0.056^+^ (2.049)	0.093^+^ (1.679)
**Additional analysis**						
PLM squared	–	–	–	–	-0.105 (-0.067)	-0.157 (-0.115)
KM capability × PLM squared	–	–	–	–	-0.213* (-2.559)	-0.214* (-2.325)
R^2^	0.260	0.331	0.342	0.435	0.368	0.349
Adjusted R square	0.234	0.302	0.319	0.408	0.337	0.323
F	9.989***	11.145***	14.730***	19.638*	15.012***	16.967***

All beta coefficients are standardized, with *t*-value in parentheses. MLM, market legitimation motivation; PLM, political legitimation motivation. ^+^*P* < 0.1; *P < 0.05; **P < 0.01 ***P < 0.001 (two-tailed test; *N* = 236).

**FIGURE 2:**
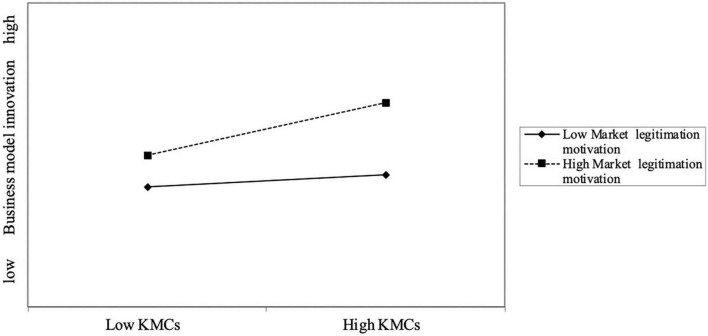
The moderation of knowledge management capabilities (KMCs)–business model innovation (BMI) relationship by political legitimation motivation (PLM).

Based on Model 2, MLM and PLM both are significantly related KMCs (β = 0.308, *p* < 0.001; β = 0.130, *p* < 0.05). The explanatory power (adjusted R2) in KMCs is 30.2%. Consequently, the research result supports H1 and H2, which means the degree of PLM and MLM both have positive effect on the degree of KMCs. This finding empirically reiterated the importance of the legitimation motivation in the development of KMCs. In H3 and H4, we posited that both MLM and PLM would strengthen the positive relationship between KMCs and BMI. Therefore, we further entered the interaction term (KMCs and MLM, KMCs and PLM) into the main effects model following the method in the prior research (Cohen and Cohen, 2003). Results of Model 4 indicated that the linear interaction of KMCs and MLM exert a statistically significant influence on BMI (β = 0.136, *p* < 0.05), suggesting that MLM also act an important boundary conditions in the effect of KMCs on BMI. To gain further insights into the interaction relationship of KMCs and MLM, we then plotted this interaction term in Figure 2. Figure 2 plots the moderation at high (+1 SD) and low (-1 SD) level. As shown in Figure 2, the highest level of BMI is observed for high levels of both KMCs and MLM. We also calculated simple slopes of high and low levels of the moderation, and found that KMCs are related to BMI when MLM is high (*p* < 0.001) but not when it is low (*p* > 0.1). In sum, H3 is supported by our data.

The results, however, show that PLM moderation gives a small coefficient (β = 0.077) and not enough significant (*p* < 0.1), which surprisingly, contradicts with what I predicted in H4. The results reveal that higher PLM may not strengthens the positive relationship between KMCs and BMI all the time. Consequently, H4 is not supported.

### Post-hoc analysis

**FIGURE 3:**
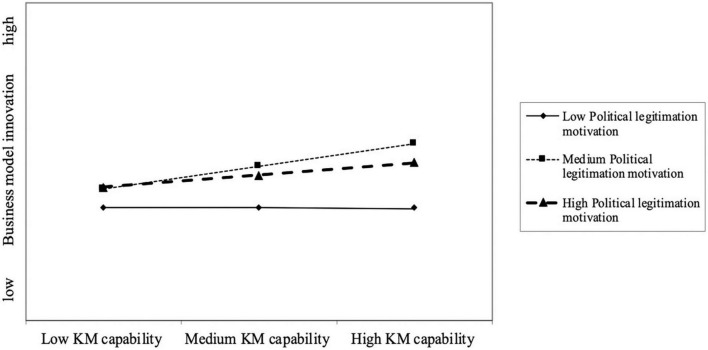
Non-linear moderation of knowledge management capabilities (KMCs)–business model innovation (BMI) relationship by political legitimation motivation (PLM).

To further explain the insignificant coefficient of the moderating effect of PLM, we conducted a post-hoc analysis to determine whether PLM had a non-linear effect on the relationship between KMCs and BMI. We believe it is helpful to clarify the potential complex influence of different motivations to achieve organizational legitimacy. Thus, we performed an additional analysis by adding the non-linear moderating effect of PLM. The results shown in Model 5 indicate that the parameter estimate of −0.213 for the product term involving a non-linear interaction (i.e., KMCs, BMI, and PLM) was statistically significant (*p* < 0.05). The explanatory power (adjusted R^2^) in BMI is 36.8%. We then plotted this interaction term in Figure 3 to better understand the curvilinear effect. The panel shows that a high level of KMCs coupled with mid-range level of PLM gives rise to a higher level of BMI of start-ups than lower or higher levels of PLM. These results show a salient inverted U-shaped effect of PLM, indicating a threshold effect. In Model 6, we included all main variables and interaction term to conduct a joint test of Hypothesis 1–4, and the results remain qualitatively unchanged. This result indicates that, differ from MLM, the PLM will posit non-linear moderating effect on the relationship between KMCs and BMI in new ventures.

## Discussion of the results

Taking the KBV and institutional theory as the foundation and based on the previously mentioned gaps in the literature, the objective of this study was to understand through what mechanism KMCs can facilitate BMI. This study combined literature on organizational legitimation, KMC and BMI, building a theoretical model to investigate the role of the motivation to achieve market and political legitimacy in developing KMCs and BMI. We proposed that the legitimation motivations play the dual role in the context of KM practices, by facilitating the KMCs as well as by shaping the these capabilities on BMI. We first tested our model by performed regression to evaluate the facilitating role of PLM and MLM on KM Cs. We then investigated the moderating role of PLM and MLM between KMCs and BMI.

Motivated by the recent calls from researchers at the interface of institutional context and KM for more empirical studies, the quantitative results first indicated that high levels of both market and political legitimation motivation are positively affect KMCs (H1 and H2). The desire to achieve legitimacy is likely to enhance collaborative relationships between internal unit and partners of market and politic, which would increase internal and external novel knowledge acquisition, sharing, and application (Maurer, 2010). This finding in line with the finding of Tsinopoulos et al. (2018), who argue that motivation linked with regulations and standards helps the organizations make better use of its relational assets to acquire and utilize the knowledge. It is also extends it by exploring different types of the role of the legitimation motivation. The findings also consistent with previous evidence reported in the literature (Lu et al., 2008; Cheng, 2010; Bunduchi, 2017). Overall, our findings are consistent with those of earlier organizational legitimacy and KM studies, hence supporting the central idea that the organizational legitimation motivation play crucial role in developing KMCs.

Furthermore, We found evidence for a contingent relationship where the influence of KMCs on BMI was highest in high level MLM (H3). the MLM had a linear moderating effect on the positive relationship between KMCs and BMI. Prior studies had argued that an important motivation for process innovation is the desire to appear legitimate to external market (Press et al., 2019). To survive or increase one’s presence in the market, firms are encourage to utilize the market experience to identity the market opportunity (Kwak et al., 2019). The significant moderation explained the effects of the motivation to achieve market legitimacy on an organizational capability to innovate. This finding indirectly validate the findings of previous studies that highlight the important effect of the motivation to achieve market legitimacy and firms innovation (Tsinopoulos et al., 2018; Kwak et al., 2019).

Interestingly, contrary to our hypothesis (H4), the positive moderating role of PLM is insignificant. We further conduct the additional analysis to clarify the effects of PLM as prior research has argue that adherence to the political norms and rules are not always beneficial to firm performance (Gu et al., 2008; Wang and Huang, 2019). The result of additional analysis shows that PLM posit a non-linear moderating effect on the positive relationship between KMCs and BMI. The explanation for the different moderating effects of PLM and MLM is related to the difference between new ventures and established firms. A high level of PLM may consume the new venture’s limited resources, thus resulting in insufficient innovation input or low efficiency of innovation (Chen et al., 2011). Such a high motivation, therefore, would leads to political embeddedness in new venture, which weak the organizational market competition and inhibit innovation. However, a high level of MLM may constrict the established firms within existing organizational routines because MLM helps established firms build R&D cooperation with the “similar” enterprise and acquire homogeneous market resource (Zimmerman and Zeitz, 2002). Different from the established firms, new ventures are not restricted by organizational inertia and are rich in entrepreneurship (Antolin-Lopez et al., 2015). Therefore, a high level of PLM may weak the BMI in a new venture, and the benefits from PLM in the new ventures is non-linear rather than linear. This explanation is in line with that of Guo et al. (2018), who argued that the relationship of political legitimacy to product innovation is inverted U-shaped in new ventures. It also extends it by exploring the moderating role of the motivation to achieve legitimacy.

## Conclusion and contributions

In the competitive business environment, BMI is now a key strategy to help firms to achieve sustainable development (Chesbrough, 2010). Therefore, in order to innovate the business model, organizations need to acquire and leverage organizational knowledge. While past research has confirmed the contribution of KMCs in the context of BMI, attention about how do the KMCs contribute to the BMI remains limited (Teece, 2018). Based on the KBV and institutional theory, this study combines literature on organizational legitimacy, KM and BMI, developing a theoretical model to investigate the direct effect of legitimation motivation on KMCs, and the moderating effect of legitimation motivation between KMCs and BMI. Evidence from 236 new ventures showed positive direct associations between PLM and KMCs, MLM, and KMCs. This implies that firms with high level of MLM or PLM, a superior KMC are more likely to achieve. In addition, MLM also strengthen the effect of KMCs and BMI while PLM had a non-linear moderating effect on the relationship between KMCs and BMI. Overall, these finding contribute to the literature on KMCs, BMI and organizational legitimacy.

### Theoretical implications

To address the research question that how do KMCs facilitate the BMI in the new venture, we contributes to previous research in several ways. To answer our research question, we first explored the implications of our findings for the KM and BMI literature. Although previous studies on BMI suggested that the KMC is an important trigger for BMI (Wu et al., 2013; Hock-Doepgen et al., 2021), the exact processes through which KMCs stimulates firms to innovate business model are still not full clear. We established that varying levels of legitimation motivation (MLM and PLM) play dual role in the link between KMCs and BMI. Specially, the motivations to achieve organizational legitimacy exert stronger direct effect on KMCs, whereas the legitimation motivations also exert moderating effect between KMCs and BMI. These findings enable us to understand better how KMCs affect BMI. By doing so, we offer our contribution to the literature by answering the question, “Under what conditions does the presence of KMCs in firms generate competitive advantage?” (Gaimon and Bailey, 2013). This complements the stream of empirical work on KMCs by focusing attention on how this concept plays out in an legitimation motivation context with a large sample of managers.

Second, The results also makes a theoretical contribution on BMI. Previous studies on BMI often regard organizational legitimacy as an antecedent (Bohnsack et al., 2014; Press et al., 2019). Less attention has been paid to the contextual role of legitimation motivation on the firm’s strategies choice of transform the business model. We acknowledge both the positive effect arguments, which suggests that the motivation to be legitimate enable the firms to gain access to resource and achieve economic success (Desai, 2018), as well as the dark side argument, which indicates that legitimation motivation may hinder the firms’ growth by increasing organizational inertia and competitive pressure (Schembera and Scherer, 2018). The inverted U-shaped moderation of the PLM proposed implies that both arguments are valid, and the relationship among KMCs, legitimation motivation, and BMI may be more complex than what previous studies have observed (Bock et al., 2012; Hock-Doepgen et al., 2021). In this way, the study’s findings help delineate the precondition of BMI literature.

Third, rather than focusing on a unidimensional view of legitimacy, the study’s classification of legitimation motivation has allowed for contrasting the efficacy of KMCs in settings with varying legitimation motivation contexts. We, thus, heed the calls for further investigation that clarifies the effect of KMCs in clearly institutional conditions (Rizzi et al., 2009). Results indicate significant differences among various motivations to achieve organizational legitimacy, underling the importance of considering the degree of the different types of legitimation motivations. Overall, this research lays down a threshold over which to elaborate the implication of the motivation to be legitimate and provides an explanation for extant ambiguities regarding the role of legitimation motivation in the new venture’s BMI and KM framework (Rao et al., 2008).

Fourth, we also contribute to previous literature through introducing an integrative view that embraces both the KBV and institutional theory. Most previous studies of BMI have been based on a single perspective, such as a KBV and institutional theory. Research on the integration of KBV and institutional theory has yet to receive full attention in innovation research. We suggest that it is necessary to integrate institutional theory into the KM and innovation literature as institutional factors are deeply involved in the development of the innovation process. In this way, we respond to the calls for integrating institutional theory as a contextual factor in the innovation framework (Jean et al., 2014; Rosenbusch et al., 2019).

### Practical implications

This study further provides important practical value for entrepreneurs in three ways. First, our first suggestion relates to the antecedent role of legitimation motivation on KMCs. The analysis reveals that firms with high MLM and PLM benefit from the KMCs. Hence, the entrepreneurs with limited market and PLM legitimation motivation may need to enhance these two motivations in order to better capture the value of the KMCs. In order to facilitate the KMCs in new ventures, entrepreneurs are advised to understand the needs of themselves, as well as to be familiar with the organizational innovation targets in order to implement effective strategy management practices.

Second, our results indicate that the impact of KMCs and BMI is strengthened when combined with a high level of MLM. Therefore, our advice to entrepreneurs looking at KMCs as a tool to facilitate BMI in new ventures should be to pay increased attention to developing partnership with market players. Entrepreneurs should establish the motivation to follow the prevailing rules and norms in the market, and then, they are able to capture the business opportunities for BMI. In addition, the findings also show that the effect of KM capabilities on BMI in new ventures is strongest under intermediate levels of PLM but comparatively weaker when PLM is low or high. The inverted U-shaped moderating effect of PLM suggests that BMI could be achieved in different manners. Our advice to managers maintaining an intermediate level of PLM to leverage political opportunities and avoid inertia on innovation. The firms that innovate the BM must not overemphasize on PLM, otherwise, the firms would suffer from the impeding role played by an excessively high level of PLM.

### Limitation and future direction

The current study has several limitations and opportunities for further research. First, given the limitation of database, we only collect data using subjective data. Future research should incorporate panel data to increase the validity of these findings. Second, although our dataset covers a broad range of new ventures representing a variety of industries, care should be exercised in generalizing the results. Further studies could extrapolate these findings to other settings, incorporating different countries, industries, and or other time periods. The third relates to our measurement of the motivation to achieve legitimacy. Although our arguments are in line with the definition we provided and the legitimacy literature, the measurement cannot be argued that covered all aspects of the legitimacy. The final avenues for future studies stem from the H3. Future studies could therefore explore how different types of KMCs affect the results, and then compare the results with the firms in established firms.

## Data availability statement

The raw data supporting the conclusions of this article will be made available by the authors, without undue reservation.

## Author contributions

SL developed theoretical models, wrote the manuscript, and was responsible for empirical analysis. QH and JW collected and analyzed the data and participated in manuscript writing. All authors contributed to the article and approved the submitted version.

## Appendix

**Table:**

Construct and items (strongly disagree/0; strongly agree/5)
**Knowledge management capabilities. Regarding you firm, to what extent do you agree with the following statements? (CR = 0.912, α = 0.878, AVE = 0.52, MSV = 0.411)**
1. Our company creates new knowledge for application across functional boundaries. 0.775
2. Our company creates operation systems for application across functional boundaries 0.668
3. Our company creates managerial policies and processes for application across functional boundaries 0.733
4. Our company engages in the process of distributing knowledge among departments 0.710
5. Our company designs activities to facilitate knowledge sharing across functional boundaries 0.717
6. Our company engages in processes of integrating different sources of knowledge across functional boundaries 0.789
7. Our company engages in processes of transferring knowledge to employees across functional boundaries 0.715
8. Our company engages in processes which apply experiential knowledge across functional boundaries 0.763
9. Our company engages in processes which apply knowledge to solve new problems across functional boundaries 0.717
**Political legitimacy motivation: (CR = 0.832, α = 0.696, AVE = 0.62, MSV = 0.348).**
1. Importance of being authorized by the government 0.762
2. Importance of being appraised by the government 0.821
3. Importance of often being recommended by the government as industrial templates 0.785
**Market legitimacy motivation: (CR = 0.814, α = 0.689, AVE = 0.52, MSV = 0.378).**
1. Importance of being recognized by industry peers 0.769
2. Importance of being accepted by customers 0.704
3. Importance of being recognized by suppliers 0.661
4. Importance of being recognized by retailers 0.743
**Business model innovation: Please indicate your organization’s performance relative to that of the competition over the last 3 years for each of the following. (CR = 0.864, α = 0.808 AVE = 0.52, MSV = 0.359)**
1. Our business model offers new combinations of products, services and information 0.765
2. Our business model attracts a lot of new customers0.659
3. Our business model attracts a lot of new suppliers and partners 0.729
4. Our business model links participants to transactions in novel ways 0.718
5. We frequently introduce new ideas and innovations into our business model 0.678
6. Overall our business model is novel 0.752
